# Enhanced Oxytetracycline Production by *Streptomyces rimosus* in Submerged Co-Cultures with *Streptomyces noursei*

**DOI:** 10.3390/molecules26196036

**Published:** 2021-10-05

**Authors:** Tomasz Boruta, Anna Ścigaczewska

**Affiliations:** Department of Bioprocess Engineering, Faculty of Process and Environmental Engineering, Lodz University of Technology, Wolczanska 213, 90-924 Lodz, Poland; anna.kowalska.1@p.lodz.pl

**Keywords:** *Streptomyces rimosus*, oxytetracycline, co-culture, *Streptomyces noursei*, rimocidin

## Abstract

In the present study, *Streptomyces rimosus* was confronted with *Streptomyces noursei*, *Penicillium rubens*, *Aspergillus niger*, *Chaetomium globosum,* or *Mucor racemosus* in two-species submerged co-cultures in shake flasks with the goal of evaluating the oxytetracycline production and morphological development. The co-culture of *S. rimosus* with *S. noursei* exhibited stimulation in oxytetracycline biosynthesis compared with the *S. rimosus* monoculture, whereas the presence of *M. racemosus* resulted in a delay in antibiotic production. Different strategies of initiating the “*S. rimosus* + *S. noursei*” co-cultures were tested. The improvement in terms of oxytetracycline titers was recorded in the cases where *S. noursei* was co-inoculated with *S. rimosus* in the form of spores. As the observed morphological changes were not unique to the co-culture involving *S. noursei*, there was no evidence that the improvement of oxytetracycline levels could be attributed mainly to morphology-related characteristics.

## 1. Introduction

Actinomycetes representing the genus *Streptomyces* are remarkably potent bacterial producers of antibiotics [1]. A plethora of antimicrobial compounds has been isolated from *Streptomyces* so far, including streptomycin, erythromycin, rapamycin, and neomycin [2]. In this large group of structurally and functionally diverse molecules some substances have been investigated for decades, mostly in the context of biosynthetic origins, strain development, and bioprocess optimization. Many diverse strategies have been developed to improve the production of antibiotics, including the rewiring of regulatory networks through removal of repressor genes, overexpression of positive regulators, ribosome engineering (see the review of Xia et al. [3] and references therein), introduction of drug resistance-producing mutations [4], chemical and physical mutagenesis [5], genome engineering [6], and the optimization of bioprocess conditions and medium composition [7]. Several recent studies focused on the production of oxytetracycline, a broad-spectrum antibiotic, by *Streptomyces rimosus* [8,9,10,11]. This species is also known to secrete an antifungal substance rimocidin [12,13] and harbors the genetic basis for the desferrioxamine siderophore biosynthesis [14]. In a recent study, Kuhl et al. [15] demonstrated that inoculating actinobacteria into the liquid medium containing talc microparticles resulted in morphological and production-related alterations compared to the conventional cultures. Among other findings, it was shown that the microparticle-enhanced cultivation can be successfully used to improve oxytetracycline titers in the cultivation broths of *S. rimosus*. In the present work, the co-inoculation of *S. rimosus* with the spores of different species was evaluated as an alternative approach of influencing the growth and metabolic capabilities of *S. rimosus*. The idea was to comparatively evaluate the morphological and biosynthetic outcomes of *S. rimosus* co-cultivation with several different, morphologically diverse filamentous microorganisms. To broaden the perspective on the biosynthetic capabilities of *S. rimosus* in co-cultures, the relative amounts of other detected secondary metabolites (the byproducts of oxytetracycline production) were also investigated.

The main goal of the present work was to evaluate the production of oxytetracycline by *S. rimosus* in submerged co-cultures with other filamentous microorganisms in relation to the observed morphological characteristics.

## 2. Results

**Figure 1:**
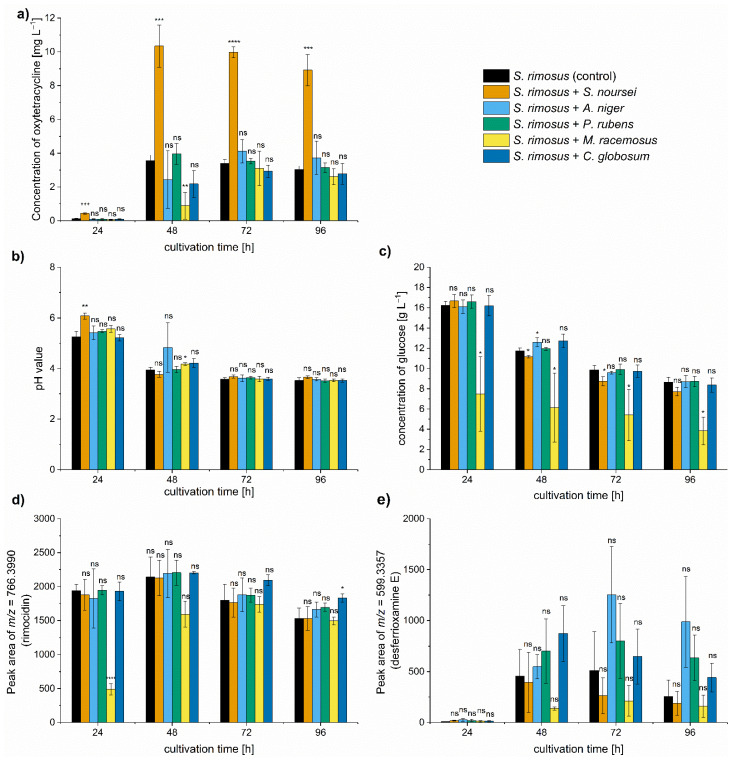
The values of oxytetracycline concentration (**a**), pH (**b**), glucose concentration (**c**), peak area corresponding to rimocidin (**d**), and desferrioxamine E (**e**) after 24, 48, 72, and 96 h of submerged co-cultivation of *S. rimosus* with *S. noursei*, *A. niger*, *P. rubens*, *M. racemosus,* or *C. globosum* in shake flasks. The inoculation with the use of spores was performed in all presented cases. The results are given as mean ± SD from three independent experiments (*n* = 3). The two-sample *t*-test was performed to indicate whether the results obtained for the co-cultures differed significantly from the ones recorded for the *S. rimosus* monoculture controls. * *p* ≤ 0.05, ** *p* ≤ 0.01, *** *p* ≤ 0.001, **** *p* ≤ 0.0001, ns—not significant. The peak areas are given in auxiliary units.

In the present study, *S. rimosus* was confronted with morphologically and biochemically diverse filamentous microorganisms in the two-species co-cultures performed under submerged conditions in shake flasks. The group of co-culture partners included three species of filamentous fungi classified as *Ascomycota*, namely *Penicillium rubens*, *Chaetomium globosum,* and *Aspergillus niger*, a fungus representing *Zygomycota*, namely *Mucor racemosus*, and *Streptomyces noursei*, a filamentous microorganism belonging to *Actinobacteria*. Most of the strains applied here were previously characterized by Kowalska et al. [16,17] in terms of their morphological development under submerged conditions. The co-cultures were characterized with respect to antibiotic production (Figure 1a), pH levels (Figure 1b), and the utilization of carbon source (glucose) (Figure 1c). Additional experimental steps involved the semiquantitative analysis of the byproducts of oxytetracycline production, namely rimocidin (Figure 1d) and desferrioxamine E (Figure 1e), identified by considering their respective *m*/*z* values in agreement with literature data. The morphological parameters (projected area, elongation, roughness, and morphology number) were determined based on microscopic observations conducted during the cultivation process (Figure S1).

The most important observation concerned the improvement of oxytetracycline production in the co-cultivation variant with *S. noursei*. The mean concentration of this antibiotic in the monoculture control reached 3.5 mg L^−1^ in 48 h of the run, whereas in the “*S. rimosus* + *S. noursei*” co-culture the value of 10.3 mg L^−1^ was recorded (Figure 1a). The remaining co-cultures also exhibited changes with respect to oxytetracycline titers, but the differences relative to the *S. rimosus* monoculture were not found to be significant (Figure 1a). It was also noted that the biosynthesis of oxytetracycline in “*S. rimosus* + *M. racemosus*” co-culture was visibly delayed compared to other tested variants (Figure 1a). The outcomes of oxytetracycline production were confronted with the measurements of pH (Figure 1b) and glucose levels in the broth (Figure 1c). After 24 h, the pH was below the value of 6 in all tested cultivations except the “*S. rimosus* + *S. noursei*” co-culture. However, starting from 72 h of the run, the pH ultimately stabilized at the level between 3.5 and 3.7 in all tested cases (Figure 1b). As far as the concentration of carbon source was concerned, it proceeded without striking differences among the variants, except for the “*S. rimosus* + *M. racemosus*” co-culture, in which glucose consumption was stimulated (Figure 1c). The next step was to investigate whether stimulating oxytetracycline biosynthesis by *S. noursei* was accompanied by enhancing the production of other secondary metabolites. The peaks at *m*/*z* = 766.3990 and *m*/*z* = 599.3357 agreed with the theoretical [M-H]^−^ ion values of rimocidin (Δ*m*/*z* = −0.0024) and desferrioxamine E (Δ*m*/*z* = −0.0048), respectively, and their relative amounts were assessed by determining the respective peak areas. No significant stimulation of rimocidin biosynthesis was revealed in “*S. rimosus* + *S. noursei*” nor in any other co-culture (Figure 1d). Its production was delayed in the presence of *M. racemosus*, which agreed with the observations made for oxytetracycline (Figure 1a). Although in the case of desferrioxamine E the variability of peak area values recorded among the independent experiments was rather high, there was no doubt that the oxytetracycline-favoring co-cultivation of *S. rimosus* with *S. noursei* did not elevate the levels of desferrioxamine E (Figure 1e).

With regard to the morphological characteristics of *S. rimosus*, the qualitative differences among the tested variants were observed in microscopic images (Figure S1a). The variability of projected area (Figure S1b), roughness (Figure S1c), elongation (Figure S1d), and morphology number (Figure S1e) values was rather high among the tested variants, as indicated by the standard deviation bars depicted in Figure S1, but in the digital image analysis of filamentous morphologies this was not a surprising behavior [16,17]. In most cases, no significant differences of morphological parameter values were recorded among the variants. Importantly, considering the results of the quantitative morphological analysis, the “*S. rimosus* + *S. noursei*” co-culture was not found to be exceptional compared to other investigated co-cultures (Figure S1). The changes of the so-called morphology number, a formula that combines several relevant morphological parameters [18], did not differ significantly among the tested variants (Figure S1e).

As an additional element of the study, the total ion chromatograms (TICs) were compared to determine if the co-cultivation led to the awakening of silent biosynthetic gene clusters in the investigated strains. However, no triggering of cryptic production pathways was found. Instead, an observation regarding the dominant role of *S. rimosus* was made when the TICs from all cultures were aligned (Figure S2). The chemical profiles of *S. rimosus*-involving co-cultures resembled the one displayed by *S. rimosus* itself (Figure S2a), while being distinct from the monocultures of its microbial partners considered in the study (Figure S2b). The chemical similarity between the cultivation broths containing *S. rimosus* was interpreted as a display of the dominance of this actinobacterium over the accompanying species. This was also confirmed by the microscopic observations of monocultures (Figure S3). If *S. rimosus* was present in the co-culture, it prevented the microbial partner from achieving its usually observed mature (in terms of size and shape) morphological form. There was, however, one exception in this respect, namely the co-cultures with *M. racemosus*. In this case, the fully developed morphological forms of *M. racemosus* (both the yeast-like and branching cells) were easily noticeable next to *S. rimosus* in the cultivation broth (Figure S1a).

**Figure 2:**
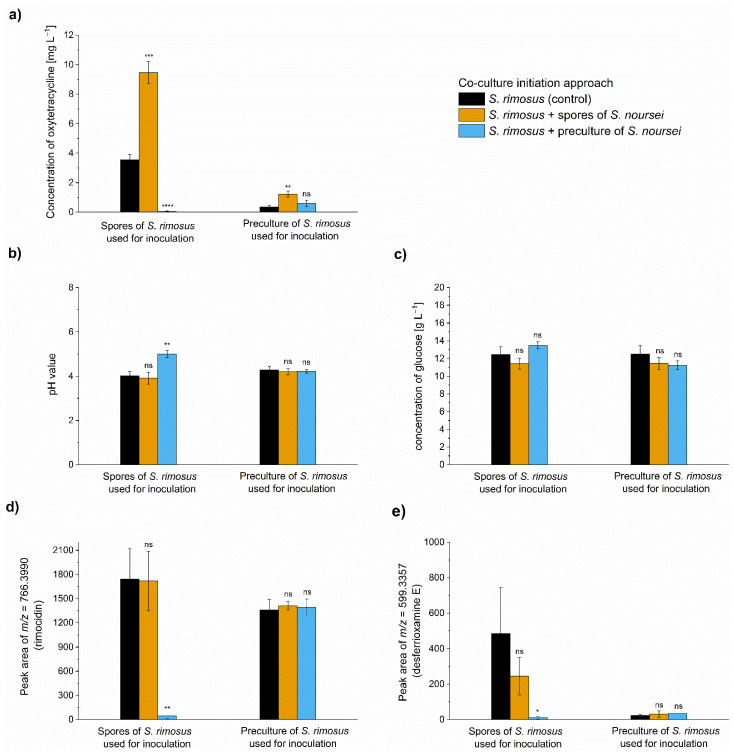
The values of oxytetracycline concentration (**a**), pH (**b**), glucose concentration (**c**), peak area corresponding to rimocidin (**d**) and desferrioxamine E (**e**) after 60 h of submerged co-cultivation of *S. rimosus* with *S. noursei* in shake flasks. Several inoculation approaches were tested. The results are given as mean ± SD from three independent experiments (*n* = 3). The two-sample *t*-test was performed to indicate whether the results obtained for the co-cultures differed significantly from the ones recorded for the *S. rimosus* monoculture controls. * *p* ≤ 0.05, ** *p* ≤ 0.01, *** *p* ≤ 0.001, **** *p* ≤ 0.0001, n—not significant. The peak areas are given in auxiliary units.

**Figure 3:**
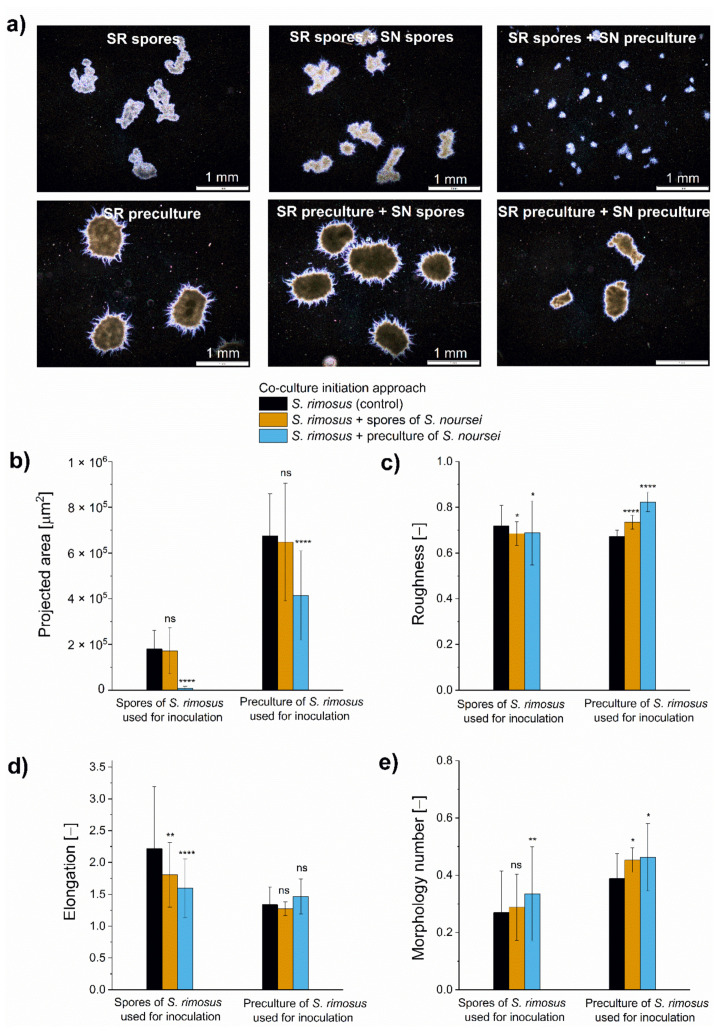
Microscopic images (**a**), projected area (**b**), roughness (**c**), elongation, (**d**) and morphology number (**e**) after 60 h of submerged co-cultivation of *S. rimosus* with *S. noursei* in shake flasks. Several inoculation approaches were tested. The results are given as mean ± SD with the average number of analyzed objects (*n*) equal to 100. The two-sample *t*-test was performed to indicate whether the results obtained for the co-cultures differed significantly from the ones recorded for the *S. rimosus* monoculture controls. * *p* ≤ 0.05, ** *p* ≤ 0.01, **** *p* ≤ 0.0001, ns—not significant, SR—*S. rimosus*, SN—*S. noursei*.

Arguably, the most frequently used approach of initiating the *Streptomyces*-involving co-cultures is to inoculate the production medium with the use of precultures (e.g., see the works reviewed in [19]). In the present study, the spores of participating species were applied instead. As the method of confronting the microorganisms in co-culture is one of the factors determining the productivity of the process, it was questioned whether the stimulatory effect of the “*S. rimosus* + *S. noursei*” co-culture would still be observed under different inoculation conditions. To address this, the spores of *S. rimosus* were used to co-inoculate the medium either with the spores or the 24 h preculture of *S. noursei* and the 60 h cultivation process was performed. In parallel, an alternative approach was tested that involved the confrontation of the 24 h preculture of *S. rimosus* with the spores or the 24 h preculture of *S. noursei*. The types of data gathered for various inoculation scenarios (“spores versus spores”, “spores versus preculture”, or “preculture versus preculture”) were the same as in the multispecies experiment described above and involved the oxytetracycline concentration (Figure 2a), pH values (Figure 2b), glucose utilization (Figure 2c), levels of rimocidin (Figure 2d), and desferrioxamine E (Figure 2e), as well as the morphological characterization (Figure 3).

The use of *S. rimosus* preculture for inoculation resulted in relatively low titers compared to the cultivations initiated from spores (Figure 2a). In both cases, however, the production of oxytetracycline was markedly improved due to the co-inoculation with the spores of *S. noursei* (Figure 2a). The stimulatory effect was visible not only when the spores of *S. noursei* were confronted with the spores of *S. rimosus*, but also in the “*S. rimosus* preculture versus *S. noursei* spores” case. Confronting the precultures of both species did not result in a significant production enhancement relative to the control. Finally, when the developed biomass of *S. noursei* was confronted with the spores of *S. rimosus*, the domination of the former took place and only trace levels of oxytetracycline were detected (Figure 2a). Hence, it was clear at this point that the co-culture initiated with the use of spores was effective in terms of improving oxytetracycline production. According to the results, however, this behavior was not associated with considerable differences in pH values (Figure 2b) nor glucose consumption (Figure 2c). It was also noted that the biosynthesis of rimocidin (Figure 2d) was less affected by the method of inoculation (preculture or spores) compared to oxytetracycline (Figure 2a) and desferrioxamine E (Figure 2e).

The morphological forms developed in the flasks inoculated with the use of *S. rimosus* precultures were generally of greater size than their counterparts in the spores-inoculated cultures (Figure 3a,b). When *S. rimosus* was confronted with *S. noursei* preculture, the presence of *S. noursei* led to a marked decrease in projected area compared to the control. This was not observed when *S. noursei* was added to the medium in the form of spores (Figure 3b). Providing *S. noursei* with a 24 h growth advantage over *S. rimosus* resulted in a clumped morphology more resembling the *S. noursei* monoculture (see Figure S3a) than the *S. rimosus* monoculture (depicted in Figure 3a). Considering the remaining morphological parameters, namely roughness (Figure 3c), elongation (Figure 3d), and morphology number (Figure 3e), it was noted that the “spores versus spores” variant, effective in the context of oxytetracycline production, did not show the morphologies that would make it stand out from other investigated cultures.

## 3. Discussion

In the present study, *S. rimosus* was co-cultivated with several diverse filamentous microorganisms under submerged conditions. Two of the tested microbial duos stood out among the tested variants, namely “*S. rimosus* + *S. noursei*” and “*S. rimosus* + *M. racemosus*”. The former turned out to be an effective combination in terms of oxytetracycline production, with one of the participating strains being able to dominate its partner from the very onset of cultivation. On the other hand, the latter pair of microorganisms could be regarded as an example of two fast-growing species that compete for nutrients and space within a limited liquid volume. This was reflected by the fact that in the early phase of the “*S. rimosus* + *M. racemosus*” co-culture the utilization of glucose was visibly stimulated compared to other tested variants (Figure 1c) due to the parallel catabolic activities of both species. Moreover, the production of oxytetracycline and rimocidin, two major secondary metabolites of *S. rimosus*, was aggravated relative to other cultures during the initial phase of the run with *M. racemosus* (Figure 1a,d). At the final days of the cultivation run, the domination of *M. racemosus* over *S. rimosus* was ultimately not observed, based on secondary metabolites production (Figure 1) and the chemical profiles reflected by the TICs (Figure S2). All in all, the clash between these “worthy microbial opponents” could not be regarded as effective in the context of elevating the levels of secondary metabolites. The issue of a producer strain being overgrown by the accompanying strain that, in principle, should only serve as a stimulating factor, was touched upon in previous efforts [20,21,22]. As the present study was focused on oxytetracycline production, the domination of *S. rimosus* over its partners in co-culture was not only not an issue, it was desired to achieve considerable production levels. In co-cultures, there was no visible inhibition exerted by *S. noursei* on *S. rimosus* in terms of the production capabilities. There was one exception, however. *S. rimosus* was not able to dominate the co-culture if it was introduced in the form of spores against the already developed biomass of *S. noursei*. The results indicated that granting the 24 h growth advantage to *S. noursei* cannot be recommended in oxytetracycline-centered cultivation.

When investigating the production of secondary metabolites in relation to morphological development, the correlations between metabolite levels and morphological characteristics are always sought. One of the behaviors typically observed in past studies involved the decrease in pellet size through morphological engineering, leading in turn to the increase in target product levels (recently reviewed in [23]). Here, when the morphological results obtained for the “*S. rimosus* versus *S. noursei*” co-cultivation were compared with the data gathered for other (less effective) co-cultures, there was no evidence that the morphology could be a key factor determining the effectiveness of this variant. It needs to be mentioned that the morphological variations recorded in the present work should be seen as rather subtle when compared with the ones found in our previous studies, e.g., when the high concentration of yeast extract resulted in the transition from the pelleted to loose morphology in *A. terreus* [24] or when the star-shaped pellets were formed in aluminum oxide-supplemented bioreactor cultures of *C. globosum* [17].

To the best of our knowledge, enhancing oxytetracycline production through co-cultivation has not been described before. However, the approach of improving secondary metabolites titers by performing microbial co-cultures was already reported. For example, undecylprodigiosin production by *Streptomyces coelicolor* was demonstrated to be boosted by the presence of *E. coli* [22]. However, the co-cultures investigated in the mentioned study were initiated by using the precultures [22], while in the present study the spores of the stimulating microorganism were applied for inoculation. This is a major difference if one considers the morphological development that occurs during the initial phases of submerged growth of pellet-forming filamentous microorganisms, most importantly the agglomeration events. If the spores of two species are confronted, there is a possibility of spores’ co-agglomeration and building a pellet around the two-species core, as was previously shown for the co-culture of *A. terreus* and *C. globosum* [25]. As a result, one of the species is “trapped” inside the pellet with its growth being inhibited by the faster-growing more aggressive dominant partner, while still exerting stimulatory effects from within the filamentous structure. In the present study, the enhancement was also observed when the “young” pellets (after 24 h of growth) of *S. rimosus* were contacted with the spores of *S. noursei*. When the pellet is developing, its structure is still relatively loose and the increase in size takes place, so the spores of the partnering species can intertwine with the biomass and become the part of the filamentous scaffold that, ultimately, becomes a structural unit responsible for generating the target product. The mechanisms of the interactions that led to the improved oxytetracycline levels were not yet deciphered and therefore are not included in this communication, but further investigation is underway. In addition, a detailed bioprocess analysis focused on the influence of medium composition, pH value, ratio of the number of spores of the participating species, process scale, and the co-cultivation initiation strategies is planned to be performed for the “*S. rimosus + S. noursei*” co-culture.

Several recent efforts addressed the regulatory and metabolic mechanisms associated with the enhanced production of antibiotics in *Streptomyces* [26,27,28]. Although in the present work, the stimulatory effect of co-cultivation was not considered in the context of molecular studies, one may speculate that the presence of *S. noursei* spores and their possible co-agglomeration with *S. rimosus* may have an influence on the oxygen levels inside the pellets and the fluxes through central carbon pathways in *S. rimosus* that provide the precursors for biosynthetic processes. In the aforementioned technique of microparticle-enhanced cultivation, where the spores of the producing microorganism form co-agglomerates with the supplied mineral particles, the altered availability of oxygen leads to marked metabolic and productivity-related consequences [29]. Furthermore, it cannot be excluded that the presence of *S. noursei* triggers a specific defense response in *S. rimosus* that involves the secretion of an antibacterial substance.

## 4. Materials and Methods

### 4.1. Strains

The following strains were used throughout the study (all purchased from the American Type Culture Collection, ATCC): *Streptomyces rimosus* ATCC 10970, *Streptomyces noursei* ATCC 11455, *Penicillium rubens* ATCC 9178, *Aspergillus niger* ATCC 204447, *Chaetomium globosum* ATCC 6205, and *Mucor racemosus* ATCC 7924. The strains were maintained on agar slants according to the recommendations of ATCC.

### 4.2. Cultivation

The commercially available ISP2 medium (Becton Dickinson, Franklin Lakes, NJ, USA) was applied to prepare the agar slants for the sporulation of *S. rimosus* and *S. noursei*. The spores of *P. rubens* and *M. racemosus* were prepared with the use of potato dextrose medium (BTL Ltd., Lodz, Poland) containing: glucose 20 g L^−1^ and potato extract 4 g L^−1^. For the sporulation of *C. globosum*, 12.5 g of commercially available pelleted rabbit food was boiled in 0.5 L of distilled water. After 30 min of steeping, agar (20 g L^−1^) was added to the filtrate. In the case of *A. niger*, the solid medium of the following composition was used for the generation of spores on agar slants: malt extract 20 g L^−1^, casein peptone 5 g L^−1^, and agar 20 g L^−1^.

The following liquid medium was used throughout the study [30]: glucose 20 g L^−1^, yeast extract 5 g L^−1^, (NH_4_)_2_SO_4_ 3 g L^−1^, KH_2_PO_4_ 2 g L^−1^, K_2_HPO_4_ 1 g L^−1^, and MgSO_4_·7H_2_O 0.5 g L^−1^. The pH was set to the value of 7 with the use of NaOH solution before autoclaving. Cultivations were carried out in flat-bottom flasks (total volume of the flask: 500 mL). To prepare the spores’ suspension of each strain, the spores were washed from agar slants to sterile medium with the use of a disposable 1 mL pipette and the number of spores was adjusted by using the Thoma chamber. For the variants inoculated with the use of spores, 150 mL of sterile medium was inoculated with 7.5 mL of spore suspension of each strain (2 strains × 7.5 mL = 15 mL in total) to achieve 10^9^ spores of a given strain per liter of medium. For the variants inoculated with the use of precultures, 150 mL of sterile medium was inoculated with 7.5 mL of each preculture (2 strains × 7.5 mL = 15 mL in total). To prepare the precultures for inoculation, 150 mL of medium was inoculated with 7.5 mL of spore suspension to achieve 10^9^ spores per liter of medium and then cultivated for 24 h. In the monoculture controls of *S. rimosus*, 150 mL of medium was inoculated with 7.5 mL of spore suspension or preculture of *S. rimosus*, and 7.5 mL of sterile medium was added instead of the spore suspension or preculture of a different microorganism. All cultivation runs were performed in an orbital shaker Certomat^®^ BS-1 (B. Braun Biotech International, Berlin, Germany) at 110 rpm and 28 °C. In the experiment involving the co-cultures of *S. rimosus* with *S. noursei*, *A. niger*, *P. rubens*, *M. racemosus,* or *C. globosum* the samples were collected every 24 h up to 96 h of the run. In the experiment testing the inoculation methods in “*S. rimosus* + *S. noursei*” co-cultures the runs were conducted for 60 h after inoculation.

### 4.3. Chemical Analysis

The filtration of the broth was performed and the filtrate was subjected to chemical analysis with the use of an ultra-high performance liquid chromatography system Acquity (Waters, Milford, MA, USA) coupled to a high-resolution mass spectrometer Synapt G2 (Waters, Milford, MA, USA), as previously described [31]. The concentration of oxytetracycline was determined with the use of a quantitative mass spectrometry-based method (under negative ionization mode, ESI^−^) developed in TargetLynx software (Waters, VMassLynx SCN781, Milford, MA, USA). The peak areas of *m*/*z* = 766.3990 and *m*/*z* = 599.3357, corresponding to the molecules identified as rimocidin and desferrioxamine E, respectively, were also determined with the use of TargetLynx (Waters, VMassLynx SCN781, Milford, MA, USA). The concentration of glucose was determined by using the Acquity system (Waters, Milford, MA, USA) equipped with an evaporated light scattering (ELS) detector and the UPLC BEH Amide (2.1 mm × 150 mm × 1.7 μm) column (75% acetonitrile solution with 0.2% triethylamine was used for elution).

### 4.4. Morphological Analysis

The microscopic observations were conducted with the use of OLYMPUS BX53 microscope (Olympus Corporation, Tokyo, Japan) as previously described [16]. The definitions of morphological parameters calculated here are provided in [16]. The size and shape of morphological forms were analyzed with the use of cellSens Dimension software (Olympus Corporation, V1.16, Tokyo, Japan).

### 4.5. Statistical Analysis

All presented results were collected during three independent experiments. In chemical analyses, the mean and standard deviation values (*n* = 3) were determined by using OriginPro (OriginLab, Vb9.4.1.354 SR1, Northampton, MA, USA). In the case of the morphological parameters, the values were extracted from the cellSens Dimension software (Olympus Corporation, V1.16, Tokyo, Japan) and the mean and standard deviation values were then determined in OriginPro, with the average number of analyzed morphological objects (*n*) equal to 100. All results were reported as “mean ± standard deviation”. The two-sample *t*-test (significance level α = 0.05) was performed to evaluate whether the values obtained for co-cultures differed significantly from the ones recorded for the *S. rimosus* monoculture controls.

## 5. Conclusions

Co-inoculation of *S. rimosus* with the spores of *S. noursei* leads to the enhancement of oxytetracycline production compared to the *S. rimosus* monoculture. By contrast, the co-cultivation with *M. racemosus* results in the delay of antibiotic production. The differences between *S. rimosus* monocultures and the corresponding co-cultures are also found at the morphological level, but there is no evidence that the morphology itself is a key factor determining the improvement of production-related performance in “*S. rimosus* + *S. noursei*” co-cultures.

## Data Availability

The data used to support the findings of this study are available from the corresponding author upon request.

## Supplementary Materials

The following are available online, Figure S1: Microscopic images of *S. rimosus* with *S. noursei*, *A. niger*, *P. rubens*, *M. racemosus,* or *C. globosum* and the values of projected area, roughness, elongation, and morphology number. Figure S2: Alignments of total ion chromatograms (TICs) obtained for the mono- and co-cultures. Figure S3: Microscopic images of monocultures.

## Sample Availability

Samples of the compounds and strains are available from the authors.
