# Supplementary material for: Enhanced Oxytetracycline Production by Streptomyces rimosus in Submerged Co-Cultures with Streptomyces noursei
Source: Molecules. 2021 Oct 5;26(19):6036. doi: 10.3390/molecules26196036 (PMC8512450; doi:10.3390/molecules26196036)
Supplement: Supplementary file 1 [file molecules-26-06036-s001.zip › molecules-1398495-supplementary.pdf]

Supplementary material

“Enhanced oxytetracycline production by *Streptomyces rimosus* through co-cultivation with *Streptomyces noursei*”

Tomasz Boruta[*], Anna Ścigaczewska

Lodz University of Technology, Faculty of Process and Environmental Engineering, Department of Bioprocess Engineering, ul. Wolczanska 213, 90-924 Lodz, Poland

[*] Corresponding author. Phone: +48 42 631 39 77; fax: +48 42 636 56 63

E-mail address: tomasz.boruta@p.lodz.pl

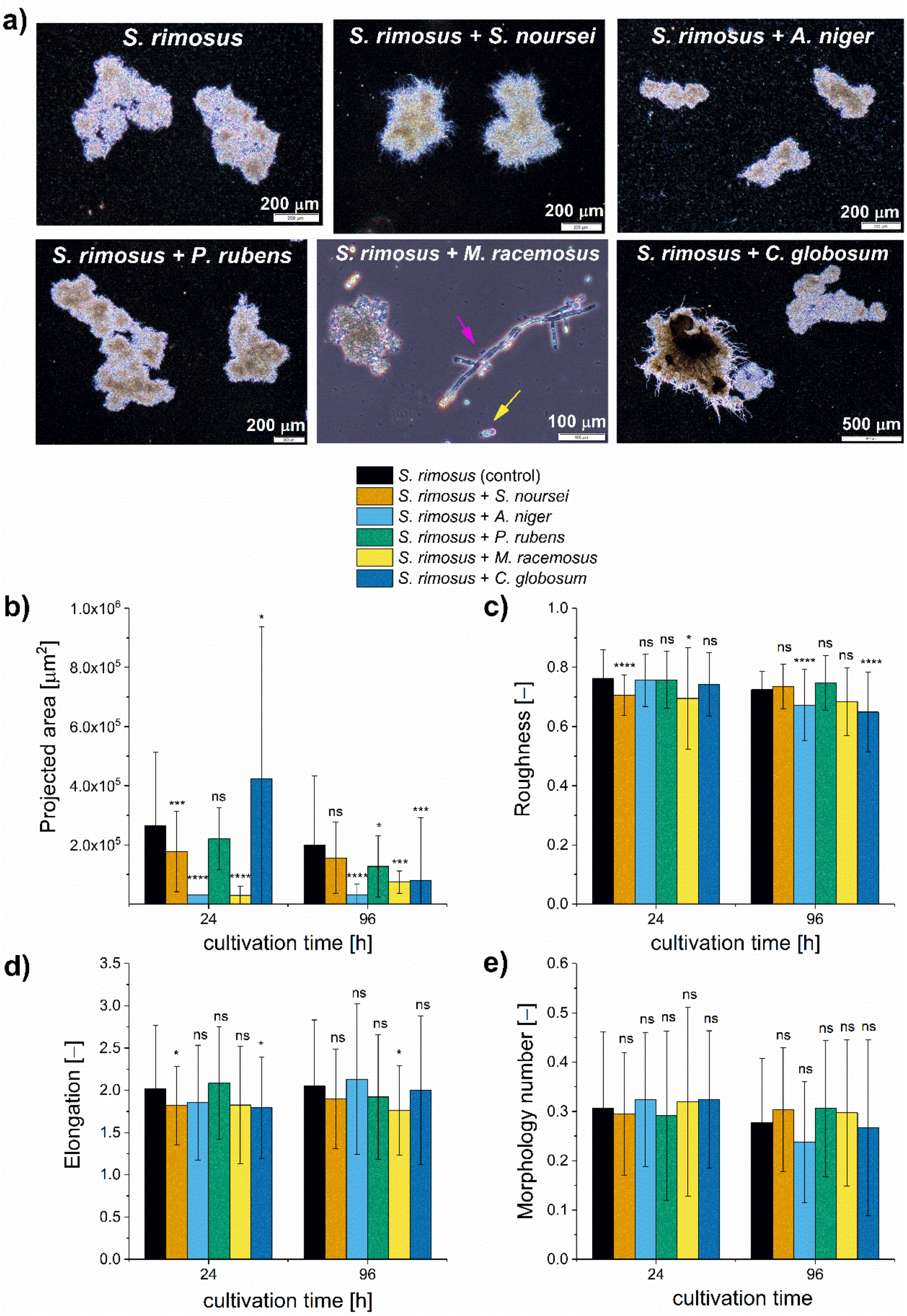

Figure S1. Microscopic images after 24 h of submerged cultivation of *S. rimosus* with *S. noursei*, *A. niger*, *P. rubens*, *M. racemosus* or *C. globosum* in shake flasks (a) and the values of projected area (b), roughness (c), elongation (d) and morphology number (e) after 24 and 96 h. The inoculation with the use of spores was performed in all presented cases. The yeast-like cells (indicated with a yellow line) and the branched hyphae (magenta line) of *M. racemosus* are indicated in (a). The results are given as mean ± SD with the average number of analyzed objects (*n*) equal to 100. The two-sample *t* test was performed to indicate whether the results obtained for the co-cultures differed significantly from the ones recorded for the *S. rimosus* monoculture controls. * $p \leq 0.05$, ** $p \leq 0.01$, *** $p \leq 0.001$, **** $p \leq 0.0001$, ns – not significant.

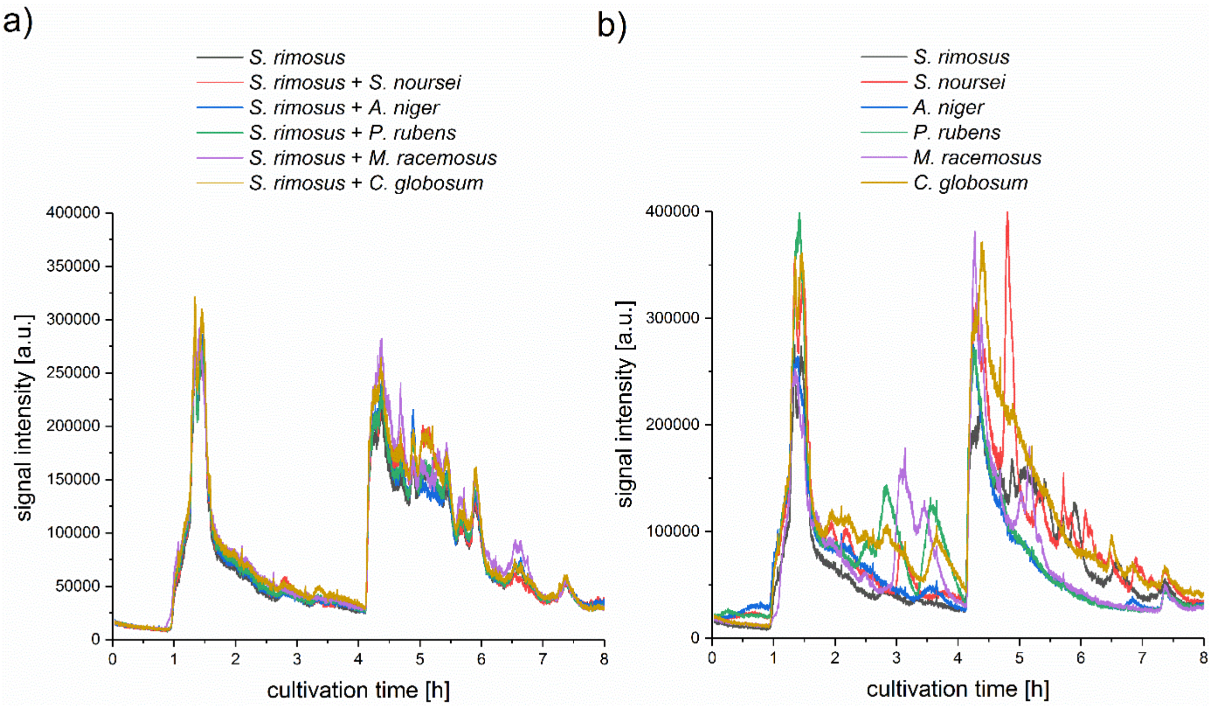

Figure S2. The total ion chromatograms aligned with (a) the co-cultures involving *S. rimosus* or (b) the monocultures of *S. noursei*, *A. niger*, *P. rubens*, *M. racemosus* and *C. globosum*. The chromatograms shown here correspond to the 72 h of cultivation. a.u. – auxiliary units.

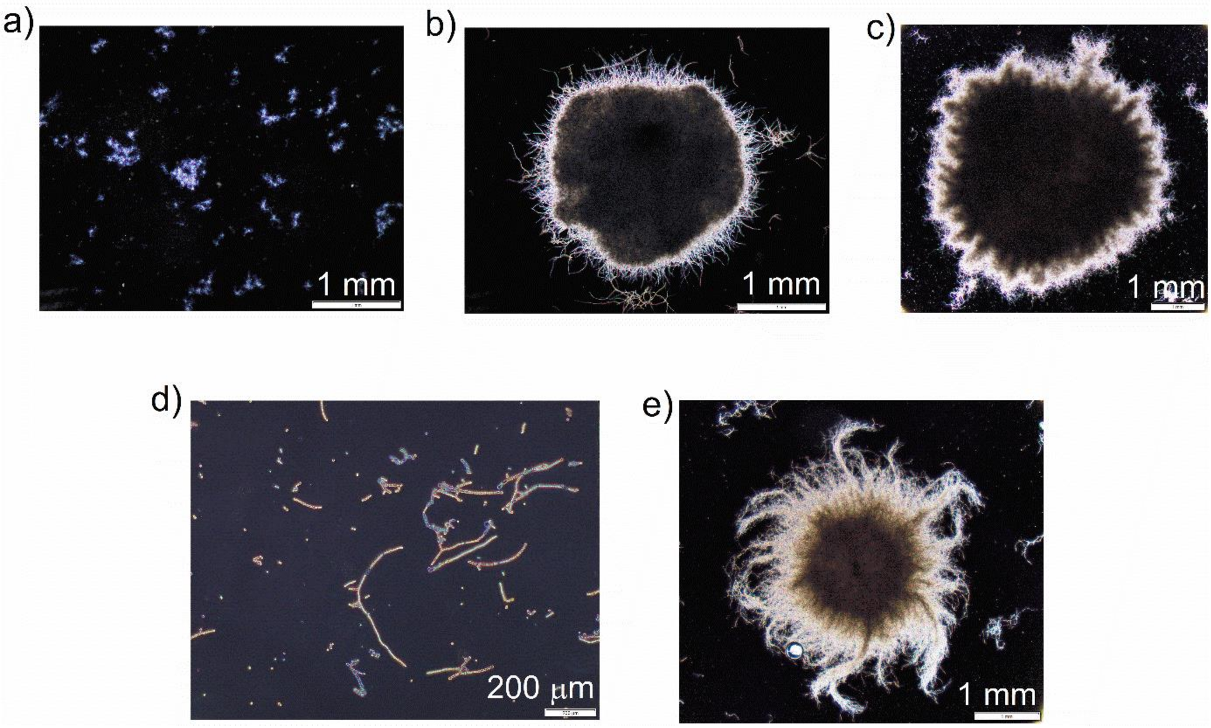

Figure S3. Microscopic images corresponding to the monocultures of (a) *S. noursei*, (b) *A. niger*, (c) *P. rubens*, (d) *M. racemosus* and (e) *C. globosum*. The images were obtained at 96 h of the cultivation run.
